# New Researches and Application Progress of Commonly Used Optical Molecular Imaging Technology

**DOI:** 10.1155/2014/429198

**Published:** 2014-02-17

**Authors:** Zhi-Yi Chen, Yi-Xiang Wang, Feng Yang, Yan Lin, Qiu-Lan Zhou, Yang-Ying Liao

**Affiliations:** ^1^Department of Ultrasound Medicine, Laboratory of Ultrasound Molecular Imaging, The Third Affiliated Hospital of Guangzhou Medical University, Guangzhou 510150, China; ^2^Department of Imaging and Interventional Radiology, Prince of Wales Hospital, Chinese University of Hong Kong, Hong Kong

## Abstract

Optical molecular imaging, a new medical imaging technique, is developed based on genomics, proteomics and modern optical imaging technique, characterized by non-invasiveness, non-radiativity, high cost-effectiveness, high resolution, high sensitivity and simple operation in comparison with conventional imaging modalities. Currently, it has become one of the most widely used molecular imaging techniques and has been applied in gene expression regulation and activity detection, biological development and cytological detection, drug research and development, pathogenesis research, pharmaceutical effect evaluation and therapeutic effect evaluation, and so forth, This paper will review the latest researches and application progresses of commonly used optical molecular imaging techniques such as bioluminescence imaging and fluorescence molecular imaging.

## 1. Introduction

Optical molecular imaging (OMI) is a molecular imaging technique developed based on genomics, proteomics, and modern optical imaging technique, through qualitative or quantitative observation and research on activities of molecule and cell in physiological and pathological processes *in vivo* with the use of specific molecular markers (such as luciferase and fluorescent protein). As one of the most powerful technology, OMI is good for viral pathogenesis researchs, immune responses to infection, and observed effects of therapy in living animals. In recent years, with the update of detecting instruments and rapid development of technologies, this noninvasive imaging technique has been increasingly widely used in disease diagnosis and therapeutic effect evaluation and some research achievements have been applied in preclinical research [1, 2]. In this paper, we will review and summarize these latest researches and application progresses of commonly used OMI techniques including bioluminescent imaging (BLI) and fluorescence molecular imaging (FMI).

## 2. Fundamental Principle

Using molecular probes and fluorescence endoscopy, the molecular signature of cells can be detection in real-time visualization with advanced targeted molecular imaging techniques [3]. OMI provides quantitative research on the distribution of fluorescent molecule and direct recording and display of biomolecule and its kinetic process, mostly absorption and scattering related organizational and biochemical information, by using some reporter genes or fluorescent dyes, and uses photon detector to detect fluorescent signal. The fluorescence emission can be spontaneously or stimulated by light with specific wavelength to appropriate fluorescent probe, or depends on protein interactions, protein degradation, and protease activity [4].

Here the reporter gene used by the OMI technology refers to protein coded by the gene that is easy to be detected, and the expression amount can reflect the regulation of target gene well. Usually products of reporter gene include firefly luciferase, green fluorescent protein (GFP), transferrin receptor, HSV1-tk, and other optimized variants applied *in vivo* with enhanced spectrum and kinetic property [5]. When reporter gene is cloned into promoter or enhancer or designed into fusion protein gene, it can monitor some basic biological processes *in vivo*, such as transcriptional control, signal transduction cascade reaction, protein-protein interaction, protein degradation, oncogene transformation, cellular transport, targeted drug effect, and so forth. Under ideal conditions, the activity of reporter gene should be consistent with the strength and duration of endogenous gene expression. Noninvasive longitudinal study can be conducted by introducing reporter gene of cell and transgenic animal [6].

With the exception of reporter gene and other bioactive substances, luminescent semiconductor nanoparticles or fluorescence dye could also be used in OMI. Quantum dot (QD), a kind of luminescent semiconductor nanoparticles, was potential for OMI as Near-infrared fluorescence imaging (NIRF) agent with deeper photon penetration [7–9].

## 3. Imaging Method

OMI includes a variety of imaging modalities such as BLI, fluorescence imaging (FI), optical coherence tomography (OCT), bioluminescent tomography (BLT), fluorescence molecular tomography (FMT), laser speckle imaging, polarization imaging, fluorescence reflectance imaging (FRI), diffuse optical tomography (DOT), fluorescence resonance imaging, NIRF, and so forth.

There are many substances that can be used for OMI, such as fluorescent protein, luciferase, fluorescent material, nanoparticle, and QD. Different OMI techniques have different imaging characteristics. For example, BLI light generation selects the substrate reaction of cell or DNA labeling with luciferase. For FI, fluorescent emission comes from protein such as GFP, red fluorescent protein (RFP), or fluorescent dye (organic fluorescent dye, nanoparticle, etc.) captured by CCD (charge coupled device) camera with a high sensitivity to monitor cell and molecular activity directly* in vivo*. For FMT, molecular probe labeled with specific fluorescent dye is injected into organism then stimulated with specific wavelength, making it emit an infrared wavelength of fluorescence longer than incident light. It can image molecules of the organism* in vivo*, which can obtain not only the distribution of tissue absorption and scattering coefficient, but also fluorescence yield and life. DOT is a near-infrared diffusion optical imaging technique imaging optical absorption and scattering coefficients of biological tissue. For NIRF, signals with different spectral characteristics are captured by camera with high sensitivity and with light of specific band as fluorescent material stimulated by light source [10, 11].

## 4. Advantages and Disadvantages

Molecular imaging is a noninvasive and real-time visualization imaging model, allowing measurement of physiological or pathological changes in the living organism at the cellular, molecular, or genetic level. Presently, five molecular imagings are available, including radionuclide imaging, X-ray computed tomography imaging (CT), magnetic resonance imaging (MRI), and ultrasound (US) imaging and OMI (Table 1) [1–3, 12–17]. The advantages of OMI are as follows: (1) characterized by high specificity and sensitivity, it can be labeled by many important genes and proteins; (2) it is suitable for long-term monitoring as OMI does not have ionizing radiation and would not cause radiation damage on organism; (3) OMI is low-cost effective, high-cost performance, and a relatively stable technology. For example, the application of GFP in molecular biology is quite mature. It can study simultaneous expression of different genes by using different molecular probes and emitting fluorescences with different wavelengths; (4) compared to other molecular imaging equipment, OMI has a relatively simple operation of imaging system; (5) OMI can measure strength (about 12 orders of magnitude), time (ranging from a few seconds to years), and size (nm to cm) and improve the temporal and spatial resolution of target cell imaging. Dynamic and quantitative study of biodynamic process can be conducted, including molecule, cell, tissue, and organism.

Disadvantages of OMI also include: (1) it is difficult to realize tomography; (2) as biological tissue is highly scattering in the range of visible light, near infrared and fluorescent signal, spatial resolution is low; (3) similar to nuclide imaging such as SPECT and PET, it is difficult to obtain the structure imaging of biological tissue; (4) due to the limitation of photon wavelength, the poor penetrating ability of photon restricts application of OMI in deep viscera *in vivo*.

## 5. Classification of Bioluminescence Imaging

According to the difference of imaging pattern, bioluminescence imaging can be divided into BLI and BLT. BLI is a high-sensitivity technique detecting cell reproduction and migration *in vivo*. The output of BLI is a two-dimensional image that optical signals gather in photondetector *in vitro* of organism. The principles of bioluminescence imaging are simple and easy to be operated, which is especially suitable for qualitative analysis and simple quantitative research. However, the accurate information of depth source with visible light in the organism cannot be obtained and it is difficult to realize accurate position of light source.

The most representative pattern of BLI is luciferase gene imaging, which is widely used and has an extremely high signal to noise ratio. It can monitor various intracellular activities such as transcription, posttranscription, translation, or posttranslation dynamically and provides a relatively simple, stable, sensitive, and cost effective way for *in vivo* imaging, and a good platform for *in vivo* biological studies [18–20]. Cool et al. [21] compared different commercially available optical imaging systems both *in vitro* and *in vivo*, which can measure both bioluminescent and fluorescent light, and they found that the IVIS Lumina II was the most sensitive system for bioluminescence imaging.

## 6. Research and Application of Bioluminescence Imaging 

### 6.1. Pharmacological Research

An ideal substrate for pharmacological research should be nontoxic and well distributed *in vivo* to be detected. Distribution and pharmacological characteristics of substrate are the most sensitive and important parameters. Therefore, it is very important to study the distribution of organism and pharmacological characteristics of the substrate. Fluorescein is a micromolecule with excellent water-solubility and lipid-solubility. It is easy to penetrate cell membrane and blood brain barrier and combine with luciferase, thus emitting bioluminescence. Therefore, it is widely used in many *in vivo* studies [22]. In the natural world, common fluoresceins include Fluc, coelenterazine, bacteriofluorescein, dinoflagellate fluorescein, and so forth. The combination of firefly luciferase and fluorescein has been successfully applied in systemic imaging. Lee et al. [23] used fluorescein analogue labeled by ^125^I to measure the biological distribution in mice without the expression of firefly luciferase. Substrate accumulation with a relatively low injected dose (<3%) was found in tissues of ossature, heart and skeletal muscle, while its accumulation in the brain was <0.5%. However, ^125^I might influence the whole biological distribution of fluorescein.

Contag et al. [24] studied the biological distribution of fluorescein by measuring luciferase in the skin of transgenic mice and found that the expression of enzyme was adjustable. The maximum response time of these transgenic mice after drug injection was 20 min. Similarly, some researchers measured the biological distribution of fluorescein in different tissues by injecting fluorescein in nontransgenic animals and using *in vitro* luciferase analysis method. Researches of Reynolds et al. [25] showed that fluorescein could be distributed all over the body without the influence of blood brain barrier or placental barrier. Though many experiments showed that substrate fluorescein could be widely distributed in systemic tissues of animal, there was no complete research on bioavailability.

Paroo et al. [26] injected 150 mg/kg or 450 mg/kg fluorescein in subcutaneously transplanted tumor nude mice and found that firefly luciferase had a stable expression. In mice with a larger dose of fluorescein injected, background luminescence increases by about 1500%, while with a standard dose, fluorescence only increases by 10%. They analyzed the strength of bioluminescence produced by systematic injection and direct intraperitoneal injection of fluorescein. The research showed that the strength of bioluminescence produced by direct injection of fluorescein was over 6 times that of intraperitoneal injection, indicating that fluorescein of intraperitoneal injection with a standard dose cannot maximize bioluminescence *in vivo*. Researches of Bhaumik and Gambhir [27] showed that imaging substrates of renilla luciferase and gossia luciferase were inferior to fluorescein. Coelenterazine was often directly injected into blood with the method of caudal vein injection or intracardiac injection. Compared to intraperitoneal injection, these methods were very complicated in imaging research. Autooxidation of coelenterazine in serum will increase background signal and it cannot pass haemal tissue barrier well, for example, blood brain barrier. In the research of virus infection, Pichler et al. [28] and Zhao et al. [29] found that the change of biological distribution of fluorescein and coelenterazine could influence the imaging effect of HSV-1. In the experimental model of corneal infection, Luker et al. [30] detected the activity of firefly luciferase after intraperitoneal injection of fluorescein. However, the luciferase activity of renilla was not detected after intravenous injection of coelenterazine. This indicated that the reasonable use of fluorescein and luciferase influences the ability of BLI detection and qualitative and quantitative researches on infectious position to a great extent.

When luciferase acts as reporter gene *in vivo*, substrate fluorescein will emit sensitive visible light in the process of oxidizing reaction. BLI technique can reduce the number of experimental animals, conduct multiple detections on the same animal at different time points, and reduce the influence of individual differences of animals on experimental data. Bioluminescence of firefly luciferase can penetrate tissues with a thickness of several centimeters. The whole progression of disease can be monitored in combination with reporter gene. Currently, it has been used for monitoring tumor growth and metastasis, infection progress of bacteria or virus, transplantation, transgenic expression, and gene therapy [31].

### 6.2. Tumor Research

Currently, BLI is most widely used in tumor research. It can observe tumor growth and metastasis of various cancer models directly and rapidly and evaluate the change in cancer therapy. Compared to MRI and PET, BLI has a high sensitivity, while PET is favorable for obtaining detection signal from a larger number of cells. BLI technique provides a noninvasive research method of dynamic biological process, such as early tumor development, therapeutic response, tumor recurrence, the evaluation of weight and position of tumor *in situ*, metastatic tumor, and spontaneous tumor, and provides a safe method for the effectiveness of monitoring *in vivo* antitumor drug.

#### 6.2.1. Research on Tumor Signal Transduction

Besides using different genes to evaluate the weight and position of tumor, BLI promotes the *in vivo* research of signal transduction process, such as reporter gene integrated with driving subarea or gene chimera sequence. When luciferase and inhibited polypeptide express in mammalian cell in the form of fusion protein, the fusion protein produced has no luciferase activity and the cell cannot give out light. However, in the case of cell apoptosis, activated caspase-3 exsects inhibited polypeptide at the specific recognition site and luciferase activity recovers. Thus, it can be used for observing the related process of *in vivo* cell apoptosis of live animals. When BLI technique is used to detect abnormal expression of receptor, the abnormal expression of epidermal growth factor receptor of kinase which is crucial to tumor growth can also be detected. Here, the optimal separation luciferase complementary system plays an important role. Each interactive protein and luciferase fragment can be integrated through the combination of EGFR and its downstream growth factor receptor binding protein 2 and Shc. After integration with interactive protein, functional luciferase can be resynthesized.

#### 6.2.2. Evaluation of Antitumor Angiogenesis Therapy

Angiogenesis is vital for tumor growth and antiangiogenesis is an important method of cancer therapy. Sanz et al. [32] implanted HUVEC and HMSC into immunodeficient mice, and monitored BLI of firefly luciferase to quantify angiogenesis. The result showed that both HSMC and transplant displayed a stable luminescent signal for up to 120 days. However, after using VEGFR tyrosine kinase inhibitor for two weeks, its significant decrease was observed using PET. It was proven that BLI could be used to evaluate the potential of drugs in antiangiogenetic therapy.

#### 6.2.3. Evaluation of Effector Cell in Tumor-Burdened Animals

It is easy to evaluate multiple immunologic functions *in vivo* by BLI technique. The research showed that effector cell survived after intravenous injection and attempts to transfer to disease area so as to keep its activity of cytotoxin *in vivo*. Effector cell migrated after passing organs such as lungs, liver, and spleen. Tumor invasion could be realized in about 3 days. To detect antineoplastic activity of these effector cells more directly, dual functional transgenosis was introduced. NK-T cell could be detected in tumor position, the time of which was 10 to 14 days, till the tumor was eradicated. The function of noninvasive research on immune cell population in disease pathogenesis and treatment reveals their key functional information in complete immune system. BLI is hopeful to be a new valuable tool for detecting *in vivo* immune response, function and relevant specific molecules.

#### 6.2.4. Establishment and Research of Tumor-Burdened Animal Model

Related gene of pulmonary metastasis of breast cancer can be studied by using BLI technique. The metastasis status and ability of different cancer cell can be evaluated by establishing a retroviral vector that can express fluorescent protein and luciferase and stably transfecting cancer cells of different subsets obtained.

**Figure 1:**
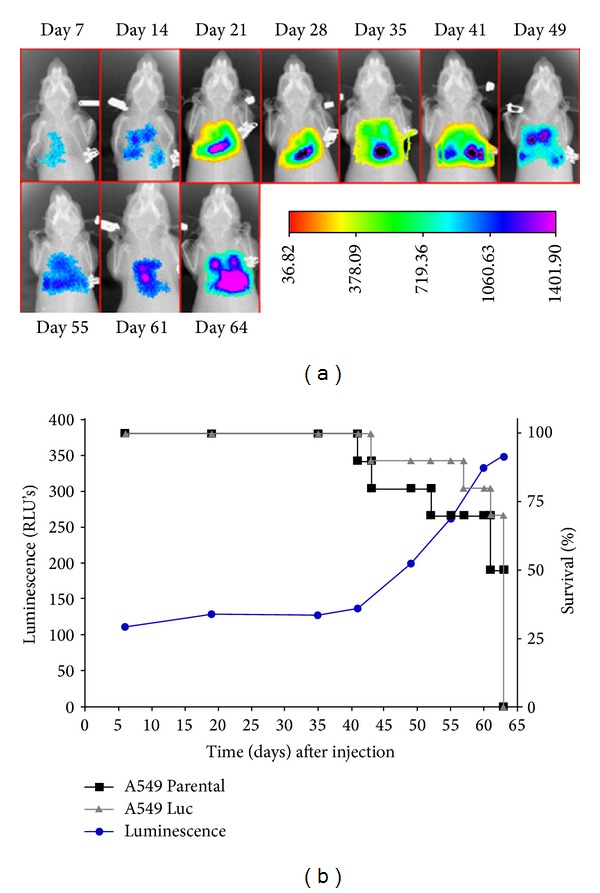
Tumor progression of orthotopical growing in nude mice with A549 Luc cancer cells. The bioluminescence activity within the thoracic cavity was monitored dynamically. (a) Up to day 64 postinoculation. (b) Luminescence alteration against time of postcell inoculation after the A549Luc cells orthotopical injection. Adapted from Madero-Visbal et al. [37].

**Figure 2:**
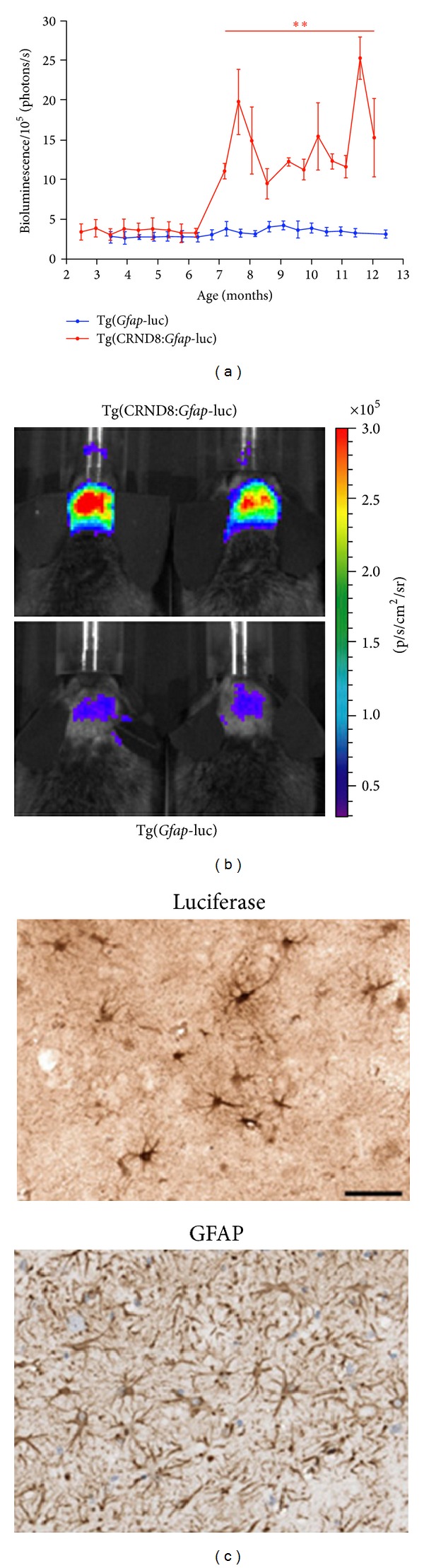
Bioluminescence imaging of disease progression in Tg(CRND8:*Gfap-*luc) mice. (a) Bioluminescence signals comparison results of Tg CRND8:*Gfap*-luc mice (red; *n* = 5) and Tg *Gfap*-luc mice (blue; *n* = 8). Tg(CRND8:*Gfap*-luc) mice showed a statistically prominent increase in BLI beginning at 7 months of age. (b) BLI signals from the brains of 12-months-old Tg(CRND8:*Gfap*-luc) mice (upper) are significant compared with age-matched Tg(*Gfap*-luc) controls (lower). (c) Immunohistochemistry on the brain of a 12-months-old Tg(CRND8:*Gfap*-luc) mouse showed prominent luciferase (upper) and GFAP (lower) staining in astrocytes within the hippocampus. Adapted from Watts et al. [40].

Podsypanina et al. [33] used breast cancer metastasis model to study whether mammary gland cell *in situ* could be induced for metastasis when there was no oncogene-driven change in the primary site. Zeng et al. [34] evaluated the therapeutic effect by the combination of rapamycin and cyclophosphamide in MDA-MB-231 nude mice transplanted tumor model and used BLI to monitor tumor growth and metastasis. The research of Kim et al. [35] proved that tracking tumor cell expressing luciferase was helpful for monitoring *in vivo* tumor metastasis and clarifying tumor metastasis mechanism. The sensitivity of tumor cell detection using BLI was close to that using PET. Stathopoulos et al. [36] studied cancer cell metastasis of lung cancer model through LLC cell labeled by GFP/luciferase reporter gene after intravenous injection of lewis lung cancer cell into the animal model. Madero-Visbal et al. [37] studied the monitoring function of BLI for nude mice model of human lung cancer and found that the average strength of bioluminescence presenting logarithmic growth and bioluminescence strength was positively correlated to tumor volume (Figure 1). Baia et al. [38] used the malignant tumor model with bioluminescence to implant modified IOMM-Lee cell in cranium of nude mice. Hashizume et al. [39] used BLI to monitor tumor growth and pharmaceutical effect and found that BLI signal and tumor volume had a good relevance in this specific area and in animal model. Watts et al. [40] used BLI to monitor the disease process of transgenic mice and found that BLI signal of transgenic mice will not enhanced with the increase of age and that bioluminescence signal of transgenic mice starts to increase significantly in the 7th month, which lasted till the end of the experiment (Figure 2).

### 6.3. The Treatment of Infectious Diseases

In infectious disease researches, the application of BLI technology cannot only be used in the observation of living site, changes in the number, and the response to external factors of pathogens in organism, but can also reveal the escape mechanism from the host defense *in vivo*. In addition, noninvasive detection of pathogen infection process contributes to revealing the latest information of disease process and the discovery of new infection sites.

**Figure 3:**
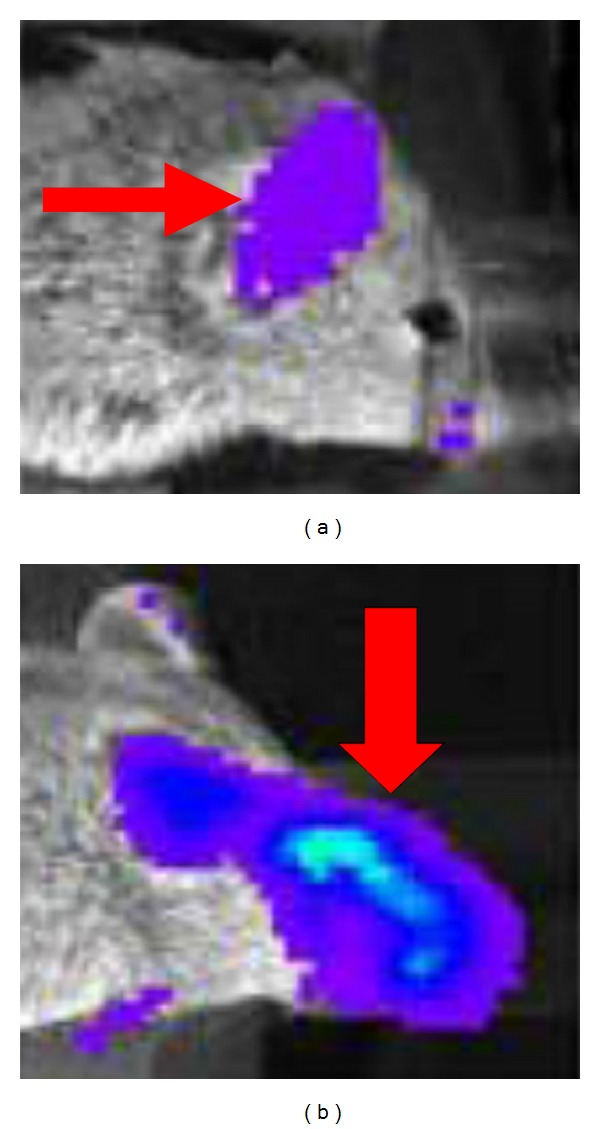
Bioluminescence imaging of HSV-1 transgenic reporter mouse. Transgenic mouse with firefly luciferase controlled by the viral promoter for HSV-1 thymidine kinase was infected with 1 × 10^6^ pfu of HSV-1 strain McKrae by corneal scarification. (a) Images prior to infection showed infection-dependent firefly luciferase bioluminescence in the infected eye and periocular tissues (red arrow). (b) Luciferase activity in the ear (red arrow) was present constitutively on day 6. Adapted from K. E. Luker and G. D. Luker [43].

**Figure 4:**
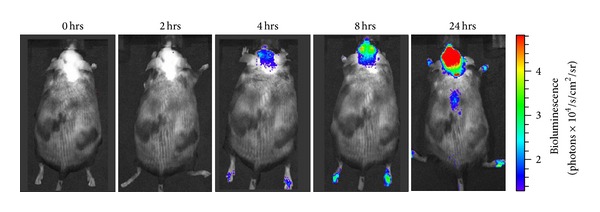
Bioluminescence imaging of transgenic C/EBP-luc reporter mice. Bioluminescent signals emitted from transgenic mice after i.p. administration of LPS (2 mg/kg). Representative images showed bioluminescence emitted from the dorsal region of the same animal in a period of 24 h. The color on the image represents the number of photons emitted from the animal per second, and a striking bioluminescent signal induced by LPS was visible in the brain region. Adapted from de Heredia et al. [47].

Contag and Bachmann [41] found that the intensity of light emissions reflected the valence of bacteria in organism. In the bacteria, homologous chromosomes recombination with genes could increase light stability of mark bacteria and could enhance gene expression. Bacteria marked with this method could be detected in the tissues of mice; the site of infection and degree could also be determined. Kadurugamuwa et al. [42] used streptococcus pneumoniae for BLI to infect transgenic mice containing firefly luciferase. K. E. Luker and G. D. Luker [43] found that ocular infection with HSV-1, firefly luciferase activity in the eye, and peri-ocular tissues increased by >10-fold above basal levels at the peak of infection on days 5-6 (Figure 3). Hwang et al. [44] used *γ*-herpes virus expressing firefly luciferase to reveal abnormal region of viral replication in salivary glands and thymus, more importantly, revealed significant increases of *γ*-herpes virus replication in lung and spleen. Sjölinder and Jonsson [45] used neisseria meningitidis to achieve real-time monitoring of bacterial sepsis in CD46 transgenic mice. It was found that the real-time BLI could become an ideal method for the longitudinal study of neisseria meningitis *in vivo*. Sadikot and Blackwell [46] established a model of pseudomonas aeruginosa pneumonia in HLL mice. After intratracheal administration of bacteria, there was an increase in chest bioluminescence that correlated with luciferase activity and neutrophilic alveolitis at 24 h. Heredia et al. [47] established a transgenic reporter mouse model, carrying the luciferase gene under the transcriptional control of C/EBP to detect the activity of C/EBP *in vivo*. Real-time BLI reflected C/EBP activity in an inflammation model, after systemic administration of lipopolysaccharides (LPS) to the C/EBP-Luc mice (Figure 4). Murphy et al. [48] investigated therapeutic effects of a small molecule *α*4-integrin antagonist in a murine model of colitis. The results indicated that BLI is a valuable strategy for preclinical evaluation of potential therapeutics that target leukocyte trafficking in inflammatory diseases.

### 6.4. The Establishment of Disease Models

Pathogenic genes, viral and bacterial, are labeled with luciferase and are transferred to animals to generate desired animal disease models. In addition to detection of the real-time expression of target genes *in vivo* and accurate response of drug candidates, the models can also be used in assessing toxicity of drug candidates and other compounds to reveal mechanism and efficacy of disease treatment.

**Figure 5:**
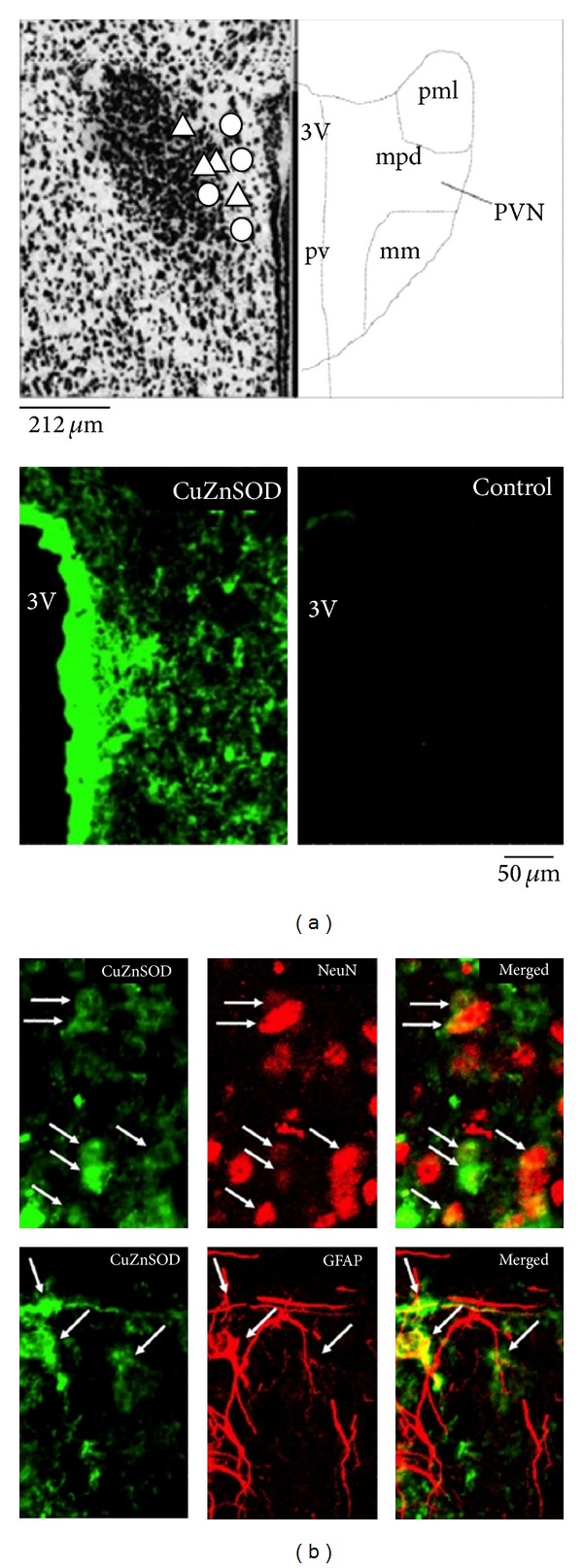
Bioluminescence imaging of regional and cellular localization of CuZnSOD in the PVN. (a) Map of the location of microinjection sites for AdLacZ (left, O) and AdCuZnSOD (left, E) in the PVN relative to the third ventricle by 4.5 mm (ventral), 0.7 mm (posterior), and 0.3 mm (lateral). Subpopulations of PVN nuclei are outlined: mm, medial magnocellular; mpd, medial parvocellular; pml, posterior magnocellular; pv, parvocellular; 3V, third ventricle. Representative photomicrographs of coronal brain sections showing CuZnSOD immunoreactivity in the PVN of AdCuZnSOD- or control-treated mice are shown on the right. (b) Representative confocal images of coronal brain sections showing CuZnSOD immunoreactivity (left), glial fibrillary acidic protein (GFAP), or neuronal nuclei (NeuN; center) and merged images (right). Adapted from Burmeister et al. [50].

Li et al. [49] injected murine embryonic stem cells carrying firefly luciferase into the hearts of mice and found that the increase in bioluminescence signal was associated with angiogenesis and improved cardiac function. Burmeister et al. [50] tested effects of excessive O_2_ and oxygen reaction regulating the activation of the redox-regulated transcription factor activator protein-1 (AP-1) in paraventricular nucleus (PVN) to the development and maintenance of renovascular hypertension. The results implicated oxidant signaling and AP-1 transcriptional activity in the PVN as key mediators in the pathogenesis of renovascular hypertension. In addition, They also found that an increase in oxide and activation of AP-1 in the PVN contributed to hypertension in 2K1C mouse model. Overexpressing CuZnSOD targeting the PVN could reduce oxide and caused a surge in AP-1 activity, and expression of a dominant-negative inhibitor of AP-1 activity in the PVN prevented 2K1C-evoked hypertension (Figure 5).

Abraham and coworkers [51] found fluctuations in bioluminescent output from the main olfactory bulbs of intact and SCN-lesioned period1-FLuc transgenic mice. Luo et al. [52] found that the vast majority of activity of Smad in SCN was positioned in pyramidal neurons in the hippocampus, indicating that BLI was a promising alternative to identify the treatment of neurons degradation diseases. Sever et al. [53] generated the ToI*β*-NOD transgenic mice in which doxycycline-inducible luciferase gene was selectively expressed in *β* cells. It was confirmed that selective expression of the luciferase gene represented a sensitive method for noninvasive monitoring of early *β*-cell dysfunction, subtle metabolic changes* in vivo*. BLI could be used in evaluation of impaired *β*-cell function in nonobese diabetic mouse model. Carlsen et al. [54] confirmed enhanced activity of luciferase in lungs or other organs when intravenous injection of endotoxin, tumor necrosis factor *α*, or interleukin-stimulation by bioluminescence and tissue luciferase measurement in NF-*κ*B mouse models. BLI could be used to track the active role of NF-*κ*B in the mouse model of lung inflammation and injury.

### 6.5. Monitoring of Gene Therapy and Gene Expression

**Figure 6:**
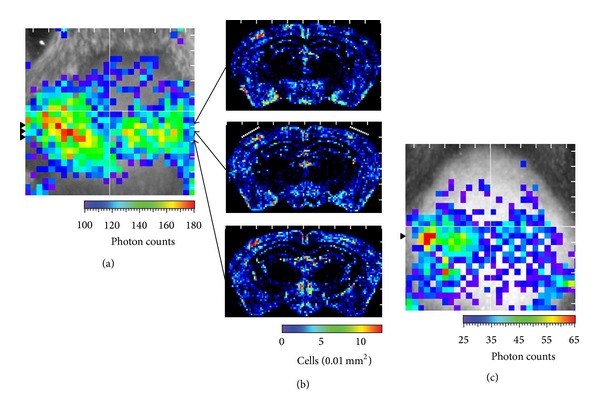
Bioluminescence imaging of transgene expression. (a) Bioluminescence. Bregma is indicated by the crossing of horizontal and vertical lines. Arrowheads show the levels of coronal sections shown in (b). (b) c-fos positive cell density maps of three coronal sections at the levels shown in (a). (c) Bioluminescence of another Fos-Luc mouse in day 40. Adapted from Wada et al. [59].

**Figure 7:**
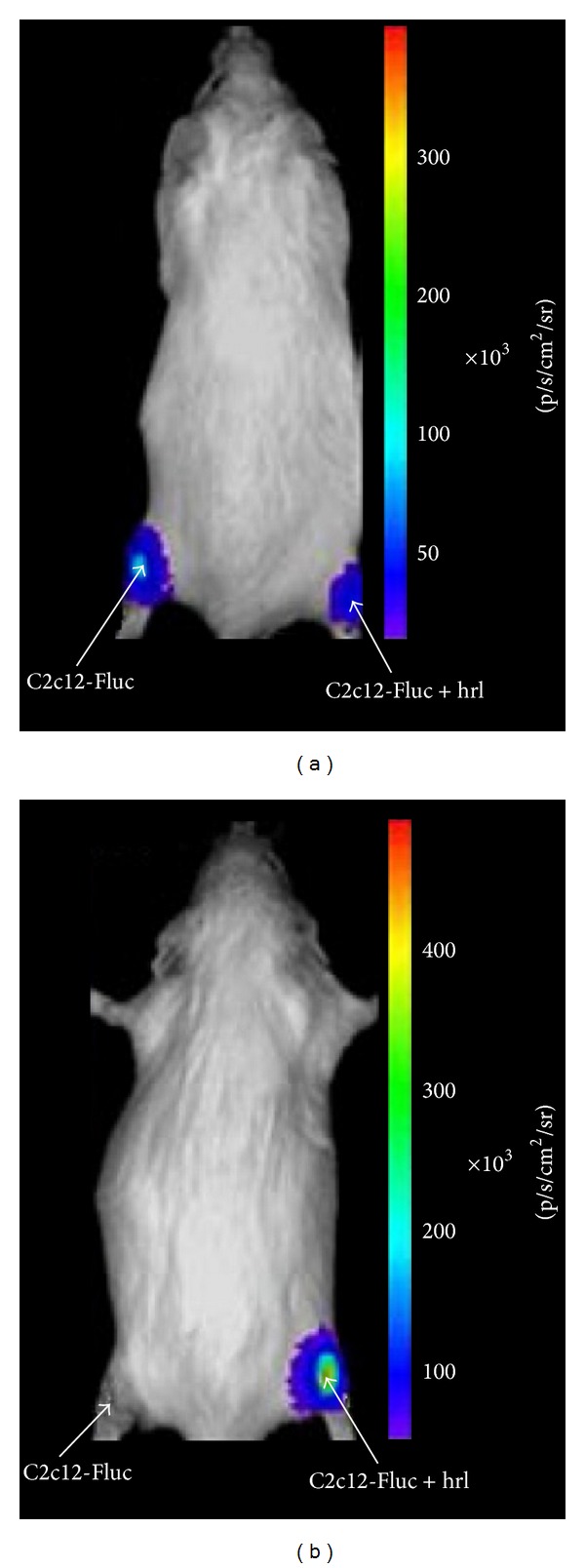
A representative bioluminescence imaging is shown demonstrating only hRL activity in the right thigh. Adapted from Gheysens et al. [60].

Vector can be used to explore the mechanisms of gene therapy and evaluate the therapeutic effect. One or multitarget genes transfected into target cells safely and effectively is expected to achieve gene therapy* in vivo*. Stirland et al. [55] used adenoviral vectors to examinate pharmacokinetics of prolactin transcription in pituitary cells. Zhang et al. [56] found that this method could also be used to measure the transcriptional regulation of hepatic cytochrome P450 enzyme (CYP3A4) in transgenic mice. Zhang et al. [57] analyzed activity of ubiquitin ligase noninvasively using a luciferase fusion protein and Cdk2 substrate. Huang et al. [58] found that hypodermic injection of plasmid encoding siRNA could be used as the effective method to induce gene silencing and improve immune activation of DNA vaccines *in vivo*. Wada et al. [59] attempted to achieve noninvasive BLI of c-fos in cerebral cortex using luciferase reporter gene. It was shown that c-fos expression could be repeatedly imaged and would not cause trauma in living animals (Figure 6). Gheysens et al. [60] injected subcutaneously C2c12-3F cells to mice and transiently transfected pUbi-hrl-Ubi-HIF-1*α*-VP2 in the left and right thigh, respectively, and both transplanted cells and HIF-1*α*-VP2 transgene expression were successfully imaged using BLI (Figure 7).

### 6.6. Others

#### 6.6.1. Studies of Embryonic Stem Cell and Regenerative Medicine

**Figure 8:**
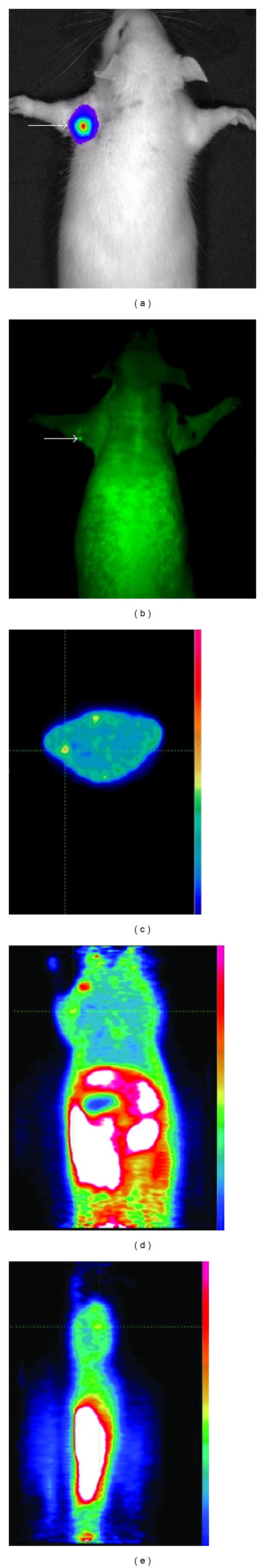
Multimodality imaging of transfected bone marrow mesenchymal stem cells. (a) Fluorescence; (b) bioluminescence; ((c), (d), (e)) micro-PET images from the transverse, coronal, and sagittal views, respectively. Clear signals in the injected site of the left upper forelimb from all three imaging modalities were observed. Adapted from Pei et al. [61].

In the field of regenerative medicine, embryonic stem cells have a great prospect. However, embryonic stem cells are injected *in vivo* and their differentiated cells have a number of disadvantages, such as significant cell death, teratoma formation, host immune rejection, and other obstacles. And mechanisms of survival, proliferation, and differentiation of embryonic stem cells after their injection into the body can be studied by BLI technology (Figure 8), so the above problems are resolved [61].

#### 6.6.2. Applications in Immunology and Organ Transplantation Biology

BLI as a valuable immunology and organ transplantation biology method not only can longitudinally track cells *in vivo*, but also can detect a range of signal transduction pathways during immune responses [62]. Nguyen et al. [63] assessed the effects of cytokines IL-18 and the role of its receptor CD30 in the developing process of GVHD by the use of a genetic defect donor or recipient mice. Nervi et al. [64] used BLI to detect the feasibility of pancreatic allograft, and to determine the best time for antilymphocyte serum treatment. It can extend graft survival and prevent graft loss. Ma and coworkers [65] demonstrated an increased NF-*κ*B activity in cardiac allograft and IRI heart transplant. Gross and Piwnica-Worms [66] confirmed that introduction of IkBa-Fluc reporter gene in liver of mice after using translated reporter gene could be used to detect ligand of *in vivo κ*B kinase (IKK), inducible inhibitor, and IKK pharmacokinetic. Blackwell and coworkers [67] found that luciferase activity of transgenic mice reflected NF-*κ*B activation of different time periods. Carlsen et al. [68] generated HLL mice to study lung and systemic NF-*κ*B-dependent inflammatory responses. HLL mouse models have proven to be valuable for measuring activation of NF-*κ*B in real-time and have helped overcome the limitation of other methods of detecting NF-*κ*B activation, such as electrophoretic mobility shift assay and western-blot analysis.

#### 6.6.3. The Application of  *In Vivo* Cell Tracking

Shah et al. [69] found that *in vivo* luminescence imaging tracer cells could also be applicable to track other cells in the body marked with luciferase reporter gene which was transplanted into mice. Thorne et al. [70] reported that some bacteria or viruses could “search for tumor” by BLI, which eliminated the need to mark tumor cells themselves and contributed to the advancement of clinical application. Invasion, migration, and expansion of tumor cells could be assessed in the model of homologous and heterologous by BLI.

#### 6.6.4. The Detection of Protein-Protein Interactions

BRET assaying protein-protein interactions of plants and animals is applicable to the study of a variety of G protein coupled receptor oligomerization [71]. Luciferase complementation imaging (LCI) is another method to detect the protein-protein interactions. In this method, two proteins were required to be tagged with complementary fragments of a luciferase [72]. Two markers have no activity when existing independently. Only after combination of two markers can they get light emitting activity. LCI can quantitatively evaluate the protein-protein interaction of a large dynamic range (1200 folds) [73]. In another application of this technology, reconstitution of renilla luciferase is applied to detect nuclear transport quantitatively. One fragment of split renilla luciferase direct at the nucleus, others direct at the test protein [74].

#### 6.6.5. Monitoring of the Biological Circadian Rhythm

One of the best ways to describe the circadian rhythm is BLI. Yamaguchi et al. [75] monitored rhythms from hundreds of single neurons for up to 11 days in SCN brain slices cultured from an mPer1: luc transgenic mouse. It was found that electrical activity played an important role in the maintenance and synchronization of single cell rhythm. Rat-1 cells and peripheral tissue explants of different mammalian have circadian rhythms of clock gene expression, which would gradually weaken after a number of cycles *in vitro*.

#### 6.6.6. Transgenic Animal Model

Models can be used to study temporal and spatial expression of specific genes in animal development and observe specific gene expression caused by drug induction and other biological processes. Chen et al. [76] found that when transcriptional regulatory sequences, transcription factors, and target genes were the same, expression level of luciferase and luminous intensity of substrates could truly reflect the expression of the target genes. Imaging studies of transgenic mice will use unmodified luciferase reporter gene. For example, unstable luciferase can be used to improve temporary resolution of infection kinetics, although these structures will reduce the overall quantity of bioluminescence [77]. Reporter gene can be activated or regulated in a tissue-specific manner in the inducible promoter mice. These studies will continue to extend the range of BLI, identify gene expression after host infection, and analyze effects of antiviral therapy.

## 7. Fluorescence Molecular Imaging

FMI mainly includes FI, FMT, OCT, FRI, DOT, and so forth, the fluorescence intensity depends not only on the nature of the fluorescent probe, but also on various parameters of the tissue, for example, the depth of fluorescent source and the density and homogeneity of the tissue. Thus, conventional fluorescent planar imaging is limited to the superficial part of the living organism and can only get semiquantitative data. FMT and DOT can obtain the 3-dimensional information of fluorescence distribution based on a correct photon propagation model that takes into consideration the absorption and divergence of both excitation and emission light. FI is usually carried out by plane or reflection imaging, the 2-dimensional image acquired by the detector is the sum of fluorescence signal emitted from the surface of the live animal. It can be rapidly, conveniently, remotely, and noninvasively obtain the overall image of a small animal.

## 8. Application of Near-Infrared Fluorescence Imaging

NIRF imaging for live animals can be used to observe the progress of cancer or other diseases as well as body responses to drug administration and can also be used in rheumatoid arthritis, rheumatoid arthritis, atherosclerosis, and thrombosis diseases.

### 8.1. Detection of Tumor

**Figure 9:**
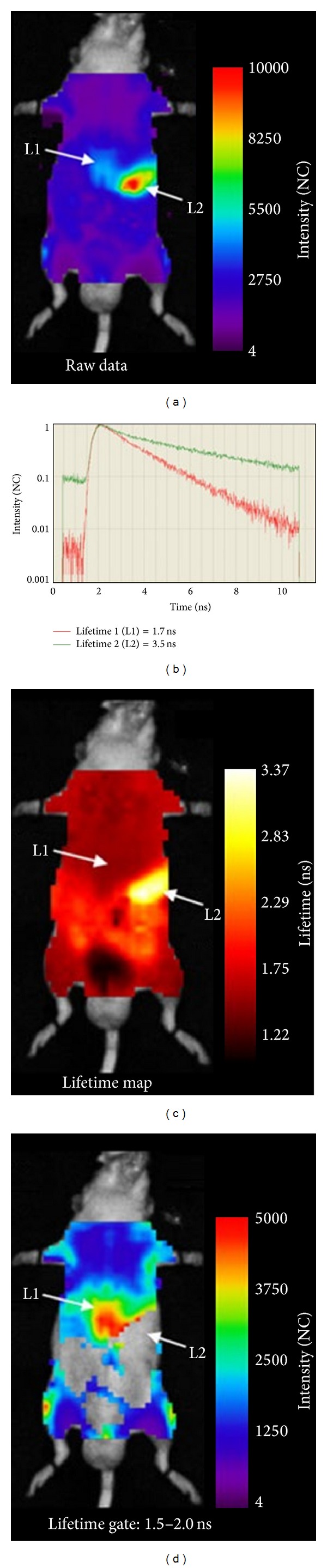
Fluorescence imaging of lifetime *in vivo*. (a) NIRF of a living mouse with a pancreatic tumour obtained 24 h after application of an antimatriptase antibody shows signals with a probespecific lifetime of 1.7 ns detectable over the tumour area (L1), as well as nonspecific signals (3.5 ns) over the stomach area (L2). (b) A representative photon time-of-flight histogram; each line corresponds to a single point on the raw scan. (c) Lifetime map displays the distribution of the different fluorescence species. (d) After gating the lifetime to 1.5–2.0 ns only specific, probe-derived fluorescence over the tumour is detectable. Adapted from Napp et al. [78].

As the ability of directly and quickly detecting the growth and metastasis of a variety of cancer models, NIRF can observe and assess cancer cells during treatment in real-time (Figure 9) [78]. NIRF can detect the primary and metastatic tumors* in vivo* noninvasively and quantitatively. The detection sensitivity is greatly improved, so that even tiny metastases can be detected (as tiny as a 10^2^ cell metastasis in the body). At present, a variety of molecular probes have been designed and synthesized the ability of early detection of tumor is efficiently improved. The imaging targets of these probes include growth-hormone-releasing hormone receptor, folate receptor, tumor cell-specific factor, calcification factor, bone formation region, calcification-bone formation region, and tumor-associated protease activating factor [79, 80].

### 8.2. Comparison of Different Tumors

The expression level of proteolytic enzymes is different in different types of tumor tissue, generally, the higher the level of aggressiveness, the higher level of proteolytic enzyme expression. NIFR can measure the expression level of the proteolytic enzymes within the organism sensitively, and different types of tumor can be distinguished by the fluorescence intensity of the probe that interacts with these enzymes. Researchers used proteolytic-enzyme-sensitive NIFR probe to observe mouse that bore a well-differentiated human mammary adenocarcinoma on the left sternum and invasive human breast cancer xenograft on the right, the results showed that NIFR probes could be used to image various tumors and higher aggressive tumors would show higher fluorescence signals [81, 82].

### 8.3. Evaluation of Molecular Therapies

To evaluate the effect and efficacy of dosage and timing of matrix metalloproteinase-2 (MMP-2) inhibitor, MMP-2-targeted fluorescence probe was used to monitor its levels. The response to small molecular inhibitor could be observed by NIFR imaging within 8 h after administration. These findings proved that smart (enzyme-activated) probes were applicable in NIFR imaging to monitor the efficacy of drugs. In the future, more probes for different types of drug targets will be developed.

### 8.4. Lymph Node Imaging

NIRF imaging has emerged as a new imaging modality to noninvasively visualize the lymphatics and assess lymphatic contractile function. Using NIRF during operations to image the sentinel lymph nodes may help achieve radical resection of tumor. Crane et al. [83] concluded that lymphatic mapping and detection of the sentinel lymph nodes in cervical cancer using NIRF imaging was technically feasible. However, the technique needs to be refined for full applicability in cervical cancer in terms of sensitivity and specificity. Moreover, Ting et al. [84] combined a novel boronate trap for F(-) with a near-infrared fluorophore. Attachment to targeting ligands enables localization by PET and NIRF. Facile incorporation of ^18^F into a NIRF probe should promote many synergistic PET and NIRF combinations.

### 8.5. Apoptosis Imaging

Apoptosis imaging is based on the close integration of annexin-V as a a phosphatidylserine exposed exposed on the cell surface and is also related to the cell's internal activation. Petrovsky et al. [85] used the near-infrared fluorescent dye, Cy5.5-labeled annexin-V, as the NIRF probe to image tumor apoptosis and may provide a nonradioactive method of measuring the antiproliferative effects of cancer chemotherapeutic regimens. Maxwell et al. [86] developed a cell-penetrating NIRF probe based on an activatable strategy to detect apoptosis-associated caspase activity for enhanced noninvasive analysis of apoptosis in whole cells and live animals. Moreover, ideal apoptosis imaging requires ligands of high affinity which should also be able to be quickly cleared in the circulation.

### 8.6. Arthritis Imaging

The highly specific smart probes used in NIRF for monitoring treatment response in rheumatoid Arthritis and rheumatic arthritis would be valuable for facilitating appropriate therapy and dosing, evaluating clinical outcome, and developing more effective drugs. Using an NIRF imaging “smart” probe, the presence and distribution of fluorescence were examined in arthritic joints of mice by noninvasive fluorescence [87]. Studies have shown that the fluorescence signal of the reporter gene could be seen at the site of arthritic joint but not in healthy surrounding tissue. Recently, Gemeinhardt et al. [88] correlated the intensity of NIRF of the nonspecific dye tetrasulfocyanine with MRI, histopathology, and clinical score. The results indicated that NIRF imaging using TSC, which was characterized by slower plasma clearance compared to indocyanine green, had the potential to improve monitoring of inflamed joints.

### 8.7. Atherosclerosis Imaging

**Figure 10:**
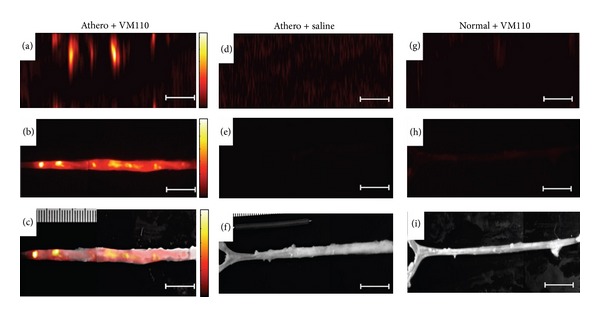
High-resolution NIRF molecular imaging of cysteine proteinase activity in atherosclerosis. (a) Aorta from atherosclerotic (athero) rabbits injected with Prosense VM110 1 day earlier and imaged with the intravascular NIRF catheter; (b) *ex vivo* fluorescence reflectance imaging (FRI) at 800 nm; (c) *ex vivo* NIRF-white light fusion image; (d) aortas from atherosclerotic rabbits injected with saline and imaged *in vivo* with the NIRF catheter; (e) *ex vivo* FRI at 800 nm; (f) *ex vivo* NIRF-white light fusion image; (g) normal rabbit injected with VM110 and imaged with the intravascular NIRF catheter; (h) *ex vivo *FRI at 800 nm (1 s) and (i) *ex vivo* NIRF-white light fusion image. Adapetd from Jaffer et al. [89].

Some proteases (cathepsin, metal matrix protease, etc.) are often highly expressed in atherosclerotic lesions. By means of probes that can be activated by proteases, NIRF can be used to monitor the formation process of atherosclerosis, providing a new tool and method for researches on the formation of hardened plaque and its frangibility (Figure 10) [89]. The Cy5.5 fluorescence dye is linked to a specific substrate of matrix metalloproteinase to form a smart probe that is activated by the enzyme. Then, this probe is used to monitor the activity of the matrix metalloproteinase in the atherosclerotic plaque in animal models. This technique provides an effective method to estimate the stage of vascular inflammation, to choose personalized treatments, and to monitor therapeutic efficacy.

### 8.8. Thrombosis Imaging

**Figure 11:**
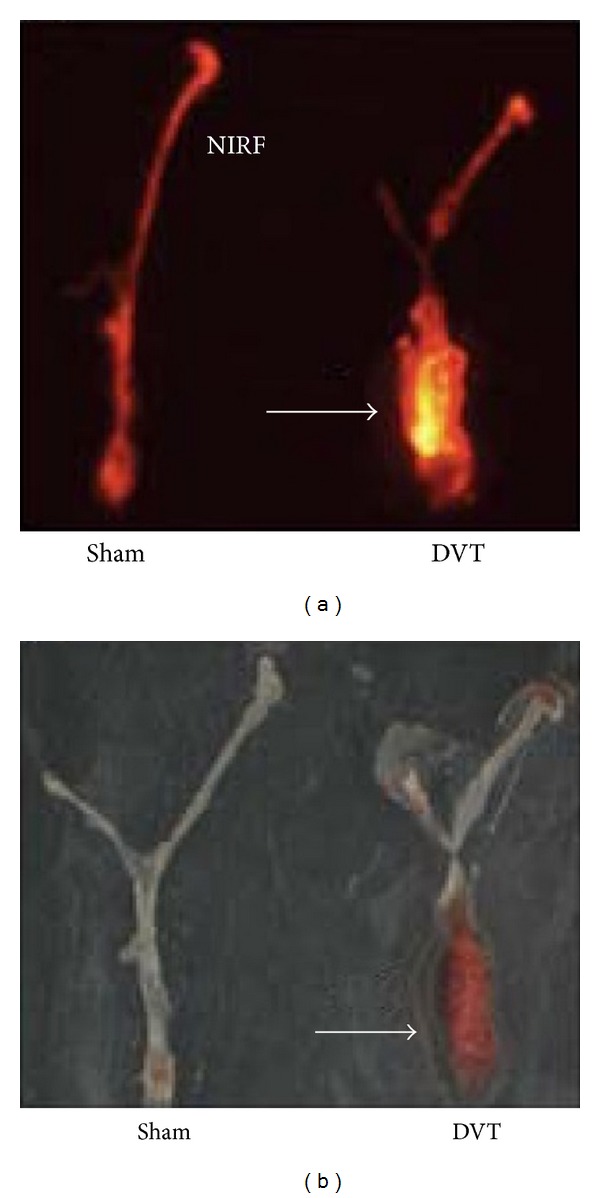
*Ex vivo* targeting of FTP11-Cy7 to thrombosed jugular veins. (a) Fluorescence reflectance image of Cy7 channel; (b) *ex vivo* image of color photograph. High NIRF signal in jugular vein was matched to the thrombosed lesion. Adapted from Hara et al. [90].

NIRF image system can be used for the image of the fiber protease rapidly for acute or chronic thrombosis, but the probe can only be injected after the formation of embolization, which is limited to the development of this technique. In recent years, researchers linked dye to a peptide of Fxllla to form a probe targeting Fxllla and used this probe to perform NIRF imaging of cerebral venous thrombosis (Figure 11) [90].

## 9. Applications of Fluorescence Molecular Imaging

### 9.1. Developmental Biology

In recent years, FMI has been widely used in detection of gene regulation and activity so as to study the cellular and molecular changes during embryonic development. Using FMI, we can intuitively observe molecular changes of cell migration and differentiation during embryonic development. Some spontaneous fluorescence proteins have been used as a reporter gene to track expression of certain genes. A family of fluorescence protein can be excited by incident light of different wavelength so that multiple molecules can be marked simultaneously. In addition, fluorescent dyes and quantum particles can also be used in these studies. FMI can also be used in transgenic detection [94, 95], and new probes for molecular imaging can be developed to monitor the transgene expression as well as the activity and function of endogenous genes. In addition, FMI can also be used to evaluate the tissue-specificity and inducibility of promoters and enhancers.

### 9.2. Cancer Research

#### 9.2.1. Tumor Growth and Metastasis

FMI can be used to observe growth and metastasis of various cancer models and to obtain a real-time image of the tumor cells so as to reveal the cellular and molecular mechanism of tumor development and changes. Naumov et al. [96] used *in vivo* video-microscopy to monitor the CHO-K1 tumor cells and detect their metastases. Yang et al. [97] transferred GFP-marked tumor cells into nude mice. In contrast to the normal tumor tissue, the newly formed tumor vessels emitted no light. According to this principle, they established a primary prostate cancer transplantation model and did further researches on quantitative analysis of angiogenesis [98]. Their results showed that vascular density of the tumor increased linearly within 10 days.

#### 9.2.2. Efficacy Evaluation of Tumor Therapy

FMI can be used to monitor changes of tumor before and after treatment of a certain drug, as well as changes of efficacy in response to dose changes of drugs. Katz et al. [99] found that irinotecan could effectively inhibit primary pancreatic tumor and metastatic tumor growth. FMI can be used for observation of the efficacy of the same drug for different subtypes of the same disease. According to the results of Schmitt et al. [100], when p53 and INK4A mutated or overexpressed or when Bcl-2 was still active, cyclophosphamide cannot induce apoptosis of lymphoma cells; FMI showed that the primary tumor and metastases progressively grew bigger in the tumor-bearing mice. While for lymphomas that had wild-type p53 and INK4A; or a silenced Bcl-2, cyclophosphamide could inhibit tumor growth by apoptosis induction, and FMI showed that the tumors significantly shrunk.

#### 9.2.3. Gene Therapy Monitoring

Through luciferase or GFP reporter gene, FMI techniques can accurately locate and quantitatively analyze gene expression and continuously track transplanted cells. In addition, spatial and temporal expression of transgenes can be monitored at the whole-organism level noninvasively and quantitatively in real time. McCaffrey et al. [101] used luciferase to label the target gene and observed a decrease in luciferase expression after siRNA and shRNA administration. For the first time, they observed that siRNA can also block expression of target genes in live animals. Using viral vectors loading luciferase or GFP, the infection activities of virus can be observed in the animal and relevant information of the infected sites can be obtained in real time by FMI.

### 9.3. Drug Research

In the field of drug research, FMI is mainly applied in drug discovery, screening, pharmacology, and small animal experiments. Biomolecular probes are used to label the drug to be tested, the labeled drug is then injected into animals, then we can monitor whether the drug reaches target area and exerts desired function. By combining imaging equipment and animal models, we can carry out rapid evaluation of novel therapies for various diseases. Combinatorial chemistry and high-throughput cell culture experiments promoted development in drug research. However, the bottleneck of drug development lies in the evaluation in animal models. High-throughput screening by FMI has incomparable advantages, such as low cost, intuitive observation, flexible probe design, and so forth. It can promote fast screening of potential therapeutic drugs, shorten preclinical studies, and accelerate drug development process. In addition, FMI technique can also detect any residual dose of certain drug quantitatively.

FMI can detect intracellular transport of certain drugs, for example, the interaction of drug-loading liposomes or microparticles with target cells and their intracellular changes. Although fluorescence microscopy is quite suitable for biological researches, it still needs to be improved in some aspects such as in the study of interactions between cationic polymers and oligonucleotides.

Drug analyses in animal models are often targeting tumor cells. Researchers have established a variety of GFP-labled primary tumor model for FMI and carried out *in vivo* screening and evaluation for antitumor growth and antiangiogenesis drugs. FMI may help reveal the cellular and molecular mechanisms of cancer development and evaluate the efficacy of anticancer drugs noninvasively. Analyses of the toxicological effects can accelerate toxicity screening procedures and improve the ability to analyze potential adverse reaction of certain drugs, thus greatly accelerating the whole process of drug development. To comprehensively understand the mechanism of certain diseases, we must conduct experiments and researches at the level of individuals. Animal models of human disease provide a good platform for this application. Traditional animal studies require a group of animals being anatomized at different time points to obtain experimental data, while *in vivo* imaging studies can observe a single object at different time points, thus avoiding deviations caused by individual variations.

## 10. Multimodal Optical Molecular Imaging

Multimodal imaging means using two or more imaging modes to observe the same object so as to obtain integrated information; both molecular and anatomical imaging data are collected, and they can complement each other, providing sufficient information for clinical diagnosis.

Deroose et al. [102] constructed a triple fusion reporter gene that enabled fluorescence-BLI-PET triple imaging for gene expression detection. Byrne et al. [103] combined BLI, PET, and MRI to study the effect of photosensitizer ADPM06 in diagnosis and treatment. First, they inoculated mice with human breast cancer cells that expressed luciferase or GFP to monitor drug distribution, metabolism, and pharmacodynamics. Then, they evaluated the efficacy of ADPM05 at 24 h/48 h after phototherapy by FDG and PET and found that tumor metabolic activity was almost completely inhibited. They used MRI to further investigate the efficacy of vascular perfusion of ADPM06.

Stem and immune cells are more frequently used as gene transferring vector, and many security issues about it can be solved by multimodal molecular imaging. Cao et al. [104] transfected mouse embryonic stem cells with a fusion reporter gene (RFP, luciferase, and HSV1-tk), evaluated cell viability noninvasively by BLI and PET imaging, and proved the effectiveness of suicide gene therapy. Waerzeggers et al. [105] used multimodal imaging for early monitoring for the stem cell gene therapy. They injected mouse NPC (expressing HSV1-tk, GFP, and Fluc) into highly malignant glioma mouse model and successfully observed by BLI not only glioma migration across the cerebral hemispheres, but also abnormal cell migration toward cerebellum and spine. These results reflected the importance of molecular imaging techniques in the study of stem and immune cells.

**Figure 12:**
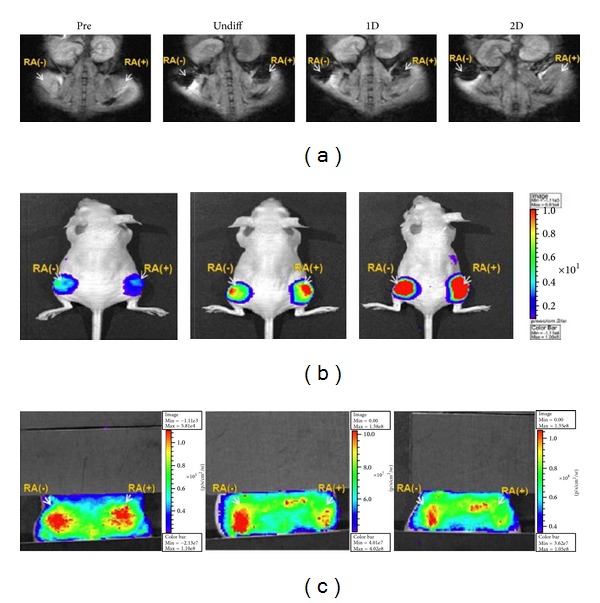
Reverse complementary multimodal imaging of miR9-involved neurogenesis of P19 cells. P19 cells cotransfected with the CMV/TfR/3XPT_miR9 and the CMV/Fluc were incorporated into a prewetted matrigel. The P19 cell-incorporated matrigels treated either without (−) or with (+) RA for neuronal induction were implanted into the right thigh (white arrow) and left thigh (white arrow) of a nude mouse, respectively (*n* = 3). MR images ((a), 1.5T), bioluminescence images, (b) and fluorescence images (c) were acquired for 2 days. MR images of the mouse bearing P19 cells without treatment of RA in the left thigh and with treatment of RA in the right thigh were acquired before and after IV injection of the MF targeting TfR nanoparticles. Adapted from Jo et al. [91].

To take advantage of the multimodal imaging systems, researches of probe that are biocompatible and integrates multiple imaging technique biocompatible for *in vivo* miRNA imaging, has attracted a lot of concern, providing complementary information about cellular and molecular activites. Jo et al. [91] developed a reverse complementary multimodal imaging system serving as a new imaging probe to image miRNA during neurogenesis using transferrin receptor (TfR) and a magnetic fluorescence nanoparticle-conjugated peptide targeting TfR (Figure 12). Multimodal imaging will become the trend in the study of miRNA expression patterns in the future. However, multimodal imaging still faces the problems of clinical transformation and biocompatibility, for example, how to genetically modify a clinical subject, possible toxicity when molecular beacons and nanoparticles are fused, and the uneven distribution of these fused particles.

**Figure 13:**
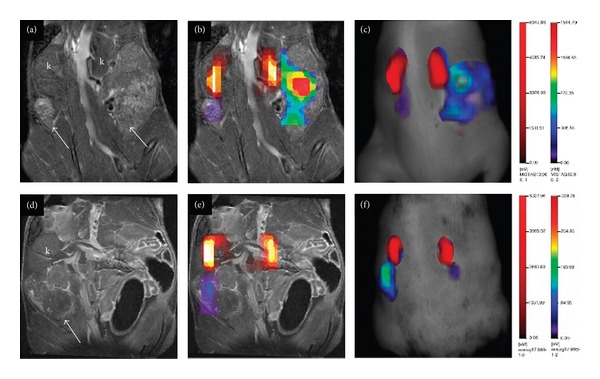
Ovarian tumors imaging with IntegriSense 680 and MMPSense 680 probes. (a) Bilateral ovarian tumors by MR imaging (arrows); (b) bilateral ovarian tumors by FMT-MR image; (c) TrueQuant images of a mouse injected systemically with IntegriSense 680 and AnnexinVivo 750; (d) MR imaging; (e) fused FMT-MR imaging; (f) TrueQuant images of a mouse injected systemically with MMPSense 680 and AnnexinVivo 750. Adapted from Hensley et al. [92].

In recent years, multimodal ultrasound contrast agents developed rapidly which include multiple parts that allow detection by a variety of techniques. It can be used not only in ultrasonic molecular imaging, but also in other imaging methods such as FMI, MR imaging, and radionuclide imaging. For example, gaseous enables ultrasound imaging; magnetic particle kernel (Fe_3_O_4_) enables magnetic resonance imaging; and when microbubbles are wrapped or connected to fluorescent molecular probes, OMI is enabled. Mulder et al. [106] prepared a dual-mode targeted paramagnetic liposome contrast agent for MRI and FMI which consisted of Gd-DTPA, fluorescent lipid, DSPC, cholesterol, and PEG-DSPE. Tumor-bearing mice were injected with *α*_v_ *β*_3_-targeting RGD-paramagnetic liposomes and nontargeting RAD-paramagnetic liposomes and were observed by MRI and fluorescence microscopy. The results showed that after intravenous injection of these contrast agents, the former was concentrated around the tumor and was limited to vascular lumen or related endothelial cells, while the latter was also concentrated to the tumor but tended to diffuse and also distributed outside the blood vessel. Hensley et al. [92] detected and quantificated tumor-associated biologic targets of epithelial ovarian cancer, and fluorescent probes were subjected to MRI and FMT, which provided quantitative information for therapy (Figure 13). Dong et al. [107] developed Gadolinium(III)-fluorescein Gd-Zpy probe which expressed a bright green fluorescence for magnetic resonance and fluorescence imaging, which was predicted to have translational applications for detection and imaging.

Nanomaterial-based contrast agents may provide a new and more effective way to achieve multimodal molecular imaging. These probes contain two or more components to allow detection by more than one imaging technique. Thus, multiple imaging techniques can complement each other to overcome their respective limitations, providing more detailed information for tissue or diseases. Veiseh et al. [108] prepared iron oxide nanoparticles for MR imaging and linked a Cy5.5 near-infrared fluorophore tag for fluorescence detection.

At present, researchers are developing different molecular imaging strategies: direct imaging probes, comparable alternative imaging probes, and transgenic animal model for reporter gene imaging. Multimodal imaging obtains images by different methods at the same time, thus integrates structural, functional, and molecular information. Different methods complement each other and thus are expected to overcome their intrinsic limitations on sensitivity and resolution. This multimodal imaging technique will have great development and application value and will become an important method of molecular imaging. This strategy combines the advantages of the different imaging techniques, forming a hybrid imaging platform superior to any single imaging technology. Various imaging strategies should closely integrate and cooperate to promote development of new imaging mode and multimodal imaging technique, so as to provide more opportunities for translational medicine research.

## 11. Prospects

As an important molecular imaging technique, OMI, especially BLI, NIRF, and FMI, has attracted remarkable attention in tumor study and drug development for its excellent performance [109]. It has the advantages of noninvasiveness, nonradiativity, high cost-effectiveness, high resolution, and simple operation in comparison with conventional imaging modalities, showing wide prospects of application in bioscience. In areas of new drug development, therapeutic assessment, and disease pathogenesis studies, this technique has already shown great advantages. Currently, OMI technology has already been widely used in various biological researches to track and rapidly evaluate the effects of various treatments; it shows great value in researches at molecular, cellular, tissular, and individual levels, holding great prospects of application in bioscience. It has already been used in the detection of gene expression and activity, determination of proteins and lipid peroxide, developmental biology and cytology assessment as well as drug research and development, study of pathogenesis, pharmacodynamic evaluation, and efficacy assessment. OMI technology has played an important role in promoting the development of molecular imaging.

**Figure 14:**
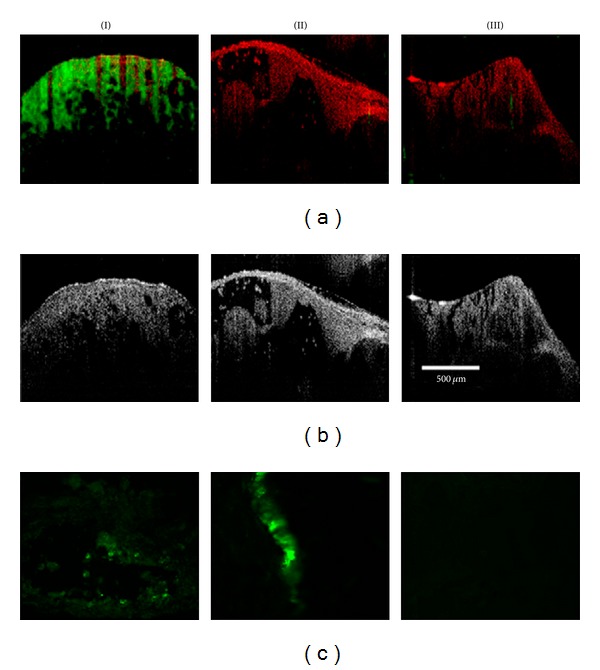
*In vivo* MM-OCT and OCT images. (a) MM-OCT signal (green channel) is superposed on the OCT signal ((b) red channel). Adapted from John et al. [93]. (c) Immunohistochemical stained sections. (I) targeted MNP-injected, (II) nontargeted MNP-injected, and (III) saline-injected rats.

OMI technology has enormous potential of applications, but it still needs to be further improved and perfected. First, contrast of tissue fluorescence against background should be further enhanced. This can be done by development of target-specific molecular probes and fluorescence tagging technique, so that the imaging probes can pass through biological barriers more easily and bind to target molecules more specifically. In addition, researches on amplification techniques and highly sensitive detection methods for specific molecular probe signals and development of new deduction algorithm may also help reduce background noise of target so as to improve accuracy. Second, deduction algorithm can be improved. The biggest challenge of OMI is the poor penetrating power of light in tissue and its absorption and scattering. Since biological tissue has the characteristics of strong scattering and low absorption, it would show a variety of optical behaviors including absorption, scattering, reflection, and transmission. Third, resolution of image needs further improvement, greatly weakening its experimental efficiency and scope of application. Using more detectors and improving existing imaging system and noise-suppressing method may help acquire image of higher resolution. Fourth, OMI should be used in combination with other imaging techniques to take advantage of multimodal and multifunction imaging, complementing each other. For instance, Magnetooptical coherence tomography (MM-OCT) that detects magnetic nanoprobes is a novel molecular imaging method stronger than contrast-enhanced imaging (Figure 14) [93].

OMI is a fast developing imaging technology, but how to convert basic researches to clinical application has always been an issue of great concern. OMI is one of the typical fields of translational medicine researches, and it has intrinsic advantages in combining basic researches and clinical studies, showing great potential for clinical application. We believe that in the near future, OMI technology will achieve a major breakthrough for the benefit of human life and health.

## Figures and Tables

**Table 1:** Comparison among different types of molecular imaging.

Type of molecular imaging	Advantage	Disadvantage
Radionuclide imaging (including PET and SPECT)	(1) To be the earliest and matureset molecular imaging technique; (2) high sensitivity and quantifiability	(1) Ionizing radiation

CT	(1) High density resolution	(1) require the ionizing radiation

MRI	(1) Highly temporal and spatial resolution; (2) flexibility; (3) no ionizing radiation; (4) simultaneous acquisition of anatomical structure and physiological function	(1) Low sensitivity

US	(1) Quantitative data, real-time practice, noninvasiveness, (2) relatively inexpensive cost, (3) no ionizing radiation, and so forth (4) as diagnostic imaging and therapeutic tool	(1) Low sensitivity

OMI	(1) Easy to use, fast, and affordable; (2) characterized by their high sensitivity; (3) labeled by many important genes and proteins; (4) causing no radiation damage on organism; (5) low cost effective, high cost performance, relatively stable technology	(1) Difficult to realize tomography; (2) near infrared and fluorescent signal, spatial resolution is low; (3) difficult to obtain the structure imaging of biological tissue; (4) the poor penetrating ability of in deep viscera *in vivo *
